# Single Cigar Price and Availability in Communities With and Without a Cigar Packaging and Pricing Regulation

**DOI:** 10.5888/pcd16.180624

**Published:** 2019-06-20

**Authors:** Lindsay Kephart, Glory Song, Patricia Henley, W.W. Sanouri Ursprung

**Affiliations:** 1 Office of Statistics and Evaluation, Massachusetts Department of Public Health, Boston, Massachusetts; 2 Massachusetts Tobacco Prevention and Cessation Program, Massachusetts Department of Public Health, Boston, Massachusetts

## Abstract

Single cigars are available for sale throughout the tobacco retail environment, are often sold for prices as low as 49 cents, and are available in flavors that appeal to youth. Since 2012, 151 municipalities in Massachusetts have enacted a minimum cigar packaging and pricing regulation that increases the price of a single cigar to a minimum of $2.50 and the price of multi-packs of 2 cigars to a minimum of $5.00. We used pricing data collected from retailers across the state to measure the effect of the regulation on price and availability of single cigars over the long term. From 2014 through 2018, the statewide average price of single cigars increased from $1.35 to $1.64, concurrent with a decrease in statewide availability. Prices of single cigars were higher in communities with the regulation but also rose over time in communities without the regulation. The increased price and decreased availability of single cigars may reduce youth exposure and access to these products.

**Summary**

**What is already known about this topic?**

Single cigars, little cigars, and cigarillos are considered starter tobacco products for youth and are available in flavors and for low prices. Localities in Massachusetts and Minnesota demonstrated that a regulation requiring a minimum price for cigars led to a short-term decrease in availability and an increase in the price of single cigars.

**What is added by this report?**

Annual pricing survey data collected from tobacco retailers in Massachusetts from 2014 through 2018 demonstrated that as more communities adopted a cigar packaging and pricing regulation, the price of single cigars increased and the availability of single cigars decreased, even in communities that had not implemented the policy. During the same time period, current youth use of cigars also decreased substantially.

**What are the implications for public health practice?**

Local municipalities who adopt similar point-of-sale tobacco regulations may contribute to a long-term increase in price and decrease in availability of single cigars among youth-accessible retailers.

## Introduction

Following the 2009 Family Smoking Prevention and Tobacco Control Act, which banned the sale of candy-flavored, fruit-flavored, and other flavored cigarettes, the largest cigarette manufacturers purchased existing cigar brands and produced cigars that were available in a variety of youth-attractive flavors, individually packaged in bright colors, and sold for as low as 49 cents each (1). From 2006 through 2010, revenue from flavored cigar sales nearly doubled among retailers in the greater Boston area, and by 2010, more than 100 different flavors of cigars were on the market (2). Data for this same period show a rise in use of cigars and cigarillos by Massachusetts youth. The retail environment is a major source of exposure and access to tobacco for youth, and policies that increase price and reduce availability of tobacco products in the retail environment are effective in curbing youth use (3).

In 2012, Boston became the first municipality in Massachusetts to implement a cigar packaging and pricing regulation (CPPR) that raises the minimum price at which single cigars or cigarillos could be sold. Studies conducted in Minneapolis and Boston demonstrated high retailer compliance with similar regulations (4,5). Ours is the first study to examine statewide single cigar price and availability of 3 cigar brands over a 5-year period.

Each year, the Massachusetts Tobacco Cessation and Prevention Program (MTCP) engages with local enforcement agents and a contracted data collection vendor to visit a large representative sample of tobacco retailers in Massachusetts and administer a survey that obtains the price and availability of different tobacco products. In odd-numbered years, the Massachusetts Youth Risk Behavior Survey (MYRBS) is administered to a representative sample of high schools in Massachusetts to collect data on youth tobacco use, including cigars. We used data from both surveys to examine single-cigar availability and price over a 5-year period in Massachusetts and statewide trends in youth cigar use during the same period.

## Purpose and Objectives

Marketing of cigars, cigarillos, and little cigars closely follows the historic pattern of tobacco industry marketing practices: use of social media, celebrity endorsements, targeted advertisements to youth and African-American populations, and increased availability in communities of color (6,7). Cigars and cigarillos are often cheaper than cigarettes, which may make them more accessible to youth, low-socioeconomic populations, and communities of color, populations all demonstrated to be price-sensitive to tobacco (8).

MYRBS surveillance data show that in 2011, high school youth’s use of cigars (14.3%) surpassed their use of cigarettes (14%) for the first time (9). Later surveys indicated that approximately 15% of youth reported that they obtained their tobacco directly or indirectly at a retail store (9).

In Massachusetts, each municipality (of 351 total) has the authority to pass health regulations, including point-of-sale tobacco control policies. CPPR requires tobacco retailers to price single cigars for a minimum of $2.50 and multi-packs of 2 or more cigars for a minimum of $5.00, although each municipality has the option to amend policy language. Violations result in tiered fines, with multiple violations resulting in permit suspension. MTCP-funded Massachusetts Board of Health programs and trade associations — Massachusetts Municipal Association, Massachusetts Association of Health Boards, and Massachusetts Health Officers Association — provide technical assistance for municipalities that consider passing tobacco control policies, including model regulation language and community mobilization at local hearings. Funded Massachusetts Board of Health programs provide retailer education and enforcement, allowing for a stable infrastructure that ensures high retailer compliance. Although some municipalities do not directly receive MTCP funds, enforcement is promoted and conducted in these municipalities, with MTCP-funded technical assistance provided by the Massachusetts Health Officers Association.

## Intervention Approach

Since Boston’s CPPR took effect in 2012, 151 municipalities in Massachusetts implemented a CPPR by the end of the study period (June 30, 2018), making up 46% of the state’s tobacco retailers and covering 47% of the state’s population. Policy passage in municipalities was as follows: 2012, n = 3; 2013, n = 30; 2014, n = 39; 2015, n = 32; 2016, n = 32; 2017, n = 12; and 2018, the end of the study period, n = 3.

State and federal policies that raise the price of cigarettes have been successful in reducing youth use of cigarettes through minimum price laws, excise taxes, minimum packaging, and the prohibition of certain flavors (10). However, lowering prices is one tactic historically used by the tobacco industry to increase demand among price-sensitive populations, including youth (11). Research has demonstrated that increases in cigarette prices have been associated with a reduction in youth use (12,13).

Like flavored cigarettes, flavored cigars have been promoted by the industry as starter products among youth, using flavors to mask the harsh tobacco taste (14). National data indicate that flavored cigars and cigarillos account for more than a third of cigar sales and half of cigarillo sales (15). A reduction in availability of single cigars may also address youth access, exposure, and use of flavored tobacco products.

## Evaluation Methods

**Pricing survey.** The pricing survey collects retailer data such as establishment name, address, store type (eg, gas station, convenience store), and whether the retailer is part of a chain or independently owned. The survey measures price and availability of 3 major cigarillo brands: Dutch Master, Black and Mild, and Garcia y Vega Game, chosen because of their prevalence in Massachusetts (2). All prices presented in this article are pre-tax prices to allow for comparison across brands.

**Pricing survey sampling.** MTCP engages with 2 groups of data collectors to conduct the pricing survey. Local enforcement agents conduct the surveys in 100% of retailers in 186 municipalities (with and without CPPR) where enforcement work is funded. In the remaining unfunded communities with at least 1 retailer present, MTCP contracts with JSI Research and Training Institute, Inc. (JSI) to perform data collection. MTCP maintains a database of all active tobacco retailers in the state from which a simple random sample of retailers in both funded and unfunded regions is drawn each quarter of the year (3-month periods). Because randomization occurs on the retailer level and not the municipal level, a representative sample of retailer data is available for each quarter throughout the year.

The study period was 5 years and collected 4 full years of data: 2014 (calendar year), and fiscal year (FY) 2016 (July 2015–June 2016), fiscal year 2017 (July 2016–June 2017), and fiscal year 2018 (July 2017–June 2018). In all years, 100% of retailers in funded municipalities were selected for surveys. For unfunded municipalities, 38% of active retailers were sampled in 2014,100% in FY 2016, 40% in FY 2017, and 100% in FY 2018, resulting in the following samples: 2014 (n = 5,471), FY 2016 (n = 6,843), FY 2017 (n = 5927), and FY 2018 (n = 4,481). Decreased sampling in 2014 and FY 2017 in unfunded communities was a result of limited funding in those years.

**Massachusetts Youth Risk Behavior Survey.** Every odd year, the Massachusetts Department of Elementary and Secondary Education and the Massachusetts Department of Public Health conduct the MYRBS to monitor trends of health risk behaviors among high school students (9). Through a random selection process, a representative sample of schools across the state is chosen to participate; within each school, classes from grades 9 to 12 are randomly selected to be surveyed. Student participation is voluntary. Surveys are administered by the Center for Survey Research at the University of Massachusetts Boston, which also prepares data for analysis, including weighting the data according to Centers for Disease Control and Prevention (CDC) protocol. Respondents are asked about their cigar use: “During the past 30 days, on how many days did you smoke cigars, cigarillos, or little cigars?” with response options that ranged from “0 days” to “all 30 days.” Respondents were considered current users if they indicated use in the past 30 days.

**Data analysis.** For each year, mean price of each brand and an aggregate mean price for all 3 cigar brands combined were calculated overall for the state and for communities with and without the CPPR. Single-cigar availability was also calculated overall for the state by individual cigar brand and aggregated for communities with and without the CPPR. Data were weighted by region and store type to account for the variation in completion rates (retailers successfully surveyed) in funded and unfunded regions, because data collectors in MTCP-funded communities are likely to have established relationships with retailers. Because of the nature of policy implementation, the CPPR within individual municipalities passed and took effect at different points over the 5 years. Individual municipalities typically provided an adequate amount of time for retailers to comply, ranging from 3 months to 1 year, so the policy effective date was used to classify whether or not a community had the regulation at the time of data collection. Communities were classified by either having a CPPR or not, despite individual variations in policy that may be present in a small subset of municipalities. At the time of this study, only aggregated numbers were available, so statistical testing or modeling could not be completed.

## Results

The average price of single cigars in Massachusetts increased steadily each year from 2014 through 2018, from $1.35 to $1.64 (Table), and availability of single cigars decreased statewide. In 2014, single cigars were available in 49% of retailers across the state. By FY 2018, single cigars were available in only 21% of retailers.

**Table:** Retailers Selling Single Cigars and Price of Cigars, Massachusetts,2014, FY2016–FY2018^a^

Variable	No. of Retailers (Average Price of Single Cigar, $)				No. of Retailers (% of Stores Selling Single Cigars)^b^			
	2014	FY 2016	FY 2017	FY 2018	2014	FY 2016	FY 2017	FY 2018
**Aggregate average^c^^,^ d ^,^ e**	7,513 (1.35)	5,842 (1.51)	3,922 (1.56)	3,794 (1.64)	7,513 (49)	5,842 (32)	3,922 (24)	3,794 (21)
Communities with no regulation	6,333 (1.17)	4,740 (1.29)	3,181 (1.35)	2,455 (1.21)	6,333 (56)	4,740 (38)	3,181 (29)	2,455 (27)
Communities with regulation	1,180 (2.24)	1,102 (2.48)	1,194 (2.50)	1,399 (2.41)	1,180 (28)	1,102 (20)	1,194 (14)	1,399 (14)
**Dutch Master**	2,583 (1.49)	1,665 (1.77)	895 (1.84)	742 (2.03)	2,583 (50)	1,665 (27)	895 (16)	742 (12)
Communities with no regulation	2,083 (1.32)	1,252 (1.53)	714 (1.68)	435 (1.70)	2,083 (55)	1,252 (30)	714 (19)	435 (14)
Communities with regulation	500 (2.50)	413 (2.50)	259 (2.50)	307 (2.45)	500 (35)	413 (22)	259 (10)	307 (10)
**Black and Mild**	2,836 (1.39)	2,812 (1.45)	2,352 (1.49)	2,716 (1.54)	2,836 (56)	2,812 (46)	2,352 (44)	2716 (44)
Communities with no regulation	2,362 (1.23)	2,308 (1.23)	1,907 (1.29)	1,788 (1.12)	2,362 (63)	2,308 (55)	1,907 (53)	1,788 (60)
Communities with regulation	474 (2.43)	504 (2.48)	707 (2.49)	928 (2.39)	474 (33)	504 (27)	707 (24)	928 (23)
**Garcia y Vega Game**	2,094 (1.00)	1,365 (1.27)	675 (1.39)	336 (1.57)	2,094 (42)	1,365 (22)	675 (13)	336 (6)
Communities with no regulation	1,888 (0.89)	1,180 (1.08)	560 (1.17)	232 (1.22)	1,888 (50)	1,180 (28)	560 (15)	232 (8)
Communities with regulation	206 (2.35)	185 (2.47)	228 (2.52)	104 (2.37)	206 (15)	185 (10)	228 (7)	104 (3)

Abbreviation: FY, fiscal year.

a 2014, calendar year; FY 2016, July 2015–June 2016; FY 2017, July 2016–June 2017; FY 2018, July 2017–June 2018. N values for each individual cigar brand (excluding the n value for the aggregate average) represent the number of retailers in the sample carrying that brand of cigars. The reduction in the N value over time is due to the reduction in the number of stores carrying single cigars as more communities across Massachusetts adopt CPPR, not a reduction in the number of retailers surveyed in the overall sample. The total number of unique retailers sampled each year is as follows: 2014 sample, n = 5,471 retailers; FY 2016 sample, n = 6,843 retailers; FY 2017 sample, n = 5,927 retailers; FY 2018 sample, n = 4,481 retailers.

b All percentages are weighted by region and store type to account for the variation in survey completion rates in funded and unfunded regions.

c The N values used for the aggregate average represent the total number of data points collected. They do not represent the number of unique stores sampled or the number of unique stores with any single cigars for sale. If a retailer carries Dutch Master, Black and Mild, and Garcia y Vega Game, it is counted 3 times.

d The aggregate average price represents the average price across all 3 cigar brands; it is calculated as: (price of all Dutch Master + price of all Black and Mild + price of all Garcia y Vega)/(total number of data points collected).

e The aggregate average percentage of retailers selling single cigars for a given year is calculated as (number of retailers that sell Dutch Master + number of retailers that sell Black and Mild + number of retailers that sell Garcia y Vega)/(total number of unique retailers sampled that year × 3).

The price of single cigars was higher in communities with the regulation than in communities without it (Table). In communities with the CPPR, the price increase of single cigars (aggregated) ranged from $2.24 to $2.41. Over time, prices of single cigars increased in communities without the regulation. The price of Garcia y Vega Game single cigars has increased from under a dollar ($0.89) to $1.22 by FY 2018 in communities without the CPPR.

Over time, availability of single cigars decreased in communities with a CPPR. From 2014 to FY 2018, availability of single cigars (aggregated) decreased from 28% to 14% in communities with the regulation. Trends over time suggest that availability of single cigars also decreased in communities without the regulation. Although availability overall for Black and Mild cigars remained steady, availability for both Dutch Master and Garcia y Vega Game single cigars dropped substantially across the state (Dutch Master, from 50% to 12%; Garcia y Vega, from 42% to 6%).

MYRBS data indicated that from 2011 through 2017, current use of cigars decreased from 14.3% to 6.7% (Figure).

**Figure:**
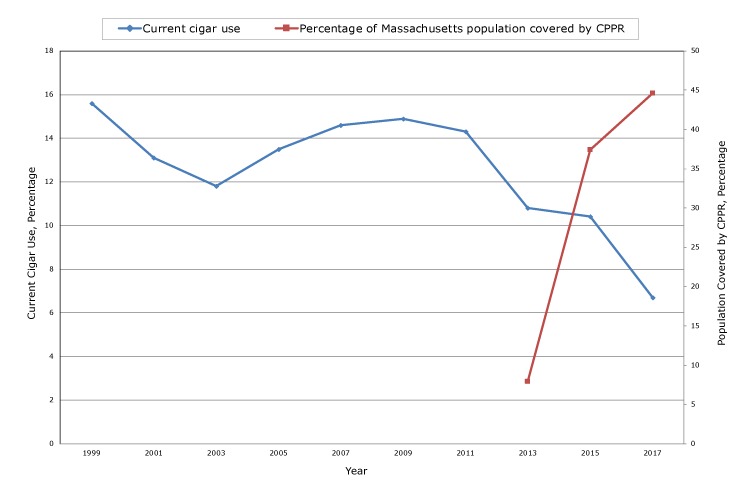
Cigar use among high school youth and percentage of population covered by cigar packaging and pricing regulation (CPPR), Massachusetts, 1999–2017. Abbreviation: NA, not applicable.

**Table:** 

Variable	1999	2001	2003	2005	2007	2009	2011	2013	2015	2017
Current cigar use, %	15.6	13.1	11.8	13.5	14.6	14.9	14.3	10.8	10.4	6.7
Population covered by CPPR,%	NA	NA	NA	NA	NA	NA	NA	7.9	37.4	44.6

## Implications for Public Health

Data for Massachusetts show an increase in the price of single cigars in several municipalities over the 5-year period. This study is the first to show that over time, with increasing policy coverage across the state, the price of single cigars increased and the availability of single cigars also decreased in communities that had not implemented the policy. The substantial statewide coverage of the CPPR may reduce youth access and youth use of cigars or cigarillos. However, other factors may affect cigar use, because youth may be switching instead to other popular nicotine products, such as e-cigarettes. Other tobacco policies passed on a municipal level, such as age restrictions, restrictions of sales of flavored tobacco products, and banning the sale of tobacco in pharmacies may also affect youth access and use.

This study has several limitations. We presented aggregated pricing and availability data, which do not allow for statistical testing; thus, we cannot directly attribute the observed outcomes to the policy. Data were unavailable before 2012, when the first CPPR was passed in Massachusetts, so we did not have a true baseline period. We used pre-tax prices for comparison purposes, and the final price may be different because of coupons or taxes. Data collection was switched from calendar year to fiscal year, leaving a gap in 2015 data. Future analysis should use individual-level retailer data to ascertain the effect of the CPPR, controlling for other tobacco control policies, community demographics, variation in policy language, and funding status.

Tobacco industry influence remains pervasive in the point-of-sale retail environment, in which youth are exposed to a variety of flavored tobacco products, advertisements, and cheap prices. A comprehensive approach to addressing tobacco industry tactics by adopting policies like the CPPR, alongside other point-of-sale policies, such as restrictions on the sale of flavored tobacco products, may increase price and reduce exposure, access, and ultimately youth use.
